# The value of the multidisciplinary team in metastatic renal cell carcinoma: Paving the way for precision medicine in toxicities management

**DOI:** 10.3389/fonc.2022.1026978

**Published:** 2023-01-12

**Authors:** Michela Roberto, Martina Panebianco, Anna Maria Aschelter, Dorelsa Buccilli, Carmen Cantisani, Salvatore Caponnetto, Enrico Cortesi, Sara d’Amuri, Claudia Fofi, Debora Ierinò, Viviana Maestrini, Paolo Marchetti, Massimo Marignani, Antonio Stigliano, Luca Vivona, Daniele Santini, Silverio Tomao

**Affiliations:** ^1^ Department of Radiological, Oncological and Anatomo-Pathological Sciences, Medical Oncology Unit A, Policlinico Umberto I Hospital, Sapienza University of Rome, Rome, Italy; ^2^ Department of Clinical and Molecular Medicine, Oncology Unit, Sant’ Andrea University Hospital, Sapienza University of Rome, Rome, Italy; ^3^ Department of Dermatology, Complex Operative Unit (UOC) of Dermatology, Policlinico Umberto I Hospital, Sapienza University of Rome, Rome, Italy; ^4^ Department of Radiological, Oncological and Anatomo-Pathological Sciences, Medical Oncology Unit B, Policlinico Umberto I Hospital, Sapienza University of Rome, Rome, Italy; ^5^ Department of Clinical and Molecular Medicine, Nephrology and Dialysis Unit, Sant’ Andrea Hospital, Sapienza University of Rome, Rome, Italy; ^6^ Department of Clinical, Internal, Anesthesiological and Cardiovascular Sciences, Sapienza University of Rome, Rome, Italy; ^7^ Scientific Direction, Istituto Dermopatico dell’Immacolata (IDI-IRCCS), Rome, Italy; ^8^ Head Liver Disease Section, Digestive and Liver Diseases Department, Sant’ Andrea Hospital, Sapienza University of Rome, Rome, Italy; ^9^ Department of Clinical and Molecular Medicine, Endocrinology Unit, Sant ‘Andrea University Hospital, Sapienza University of Rome, Rome, Italy; ^10^ Complex Operative Unit (UOC) Oncologia Medica, Sapienza University, Polo Pontino, Latina, Italy

**Keywords:** multidisciplinary team (MDT), metastatic renal cell carcinoma (mRCC), endocrinological toxicity, cardiovascular toxicity, liver toxicity, nephrological toxicity, cutaneous toxicity

## Abstract

The new landscape of treatments for metastatic clear cell renal carcinoma (mRCC) is constantly expanding, but it is associated with the emergence of novel toxicities, adding to up to those observed in the tyrosine-kinase inhibitor (TKI) era. Indeed, the introduction of immune checkpoint inhibitors (ICIs) alone or in combination has been associated with the development of immune-related adverse events (irAEs) involving multiple-organ systems which, even if rarely, had led to fatal outcomes. Moreover, due to the relatively recent addition of ICIs to the previously available treatments, the potential additive adverse effects of these combinations are still unknown. A prompt recognition and management of these toxicities currently represents a fundamental issue in oncology, since it correlates with the outcome of cancer patients. Even if clinical guidelines provide indications for the management of irAEs, no specific protocol to evaluate the individual risk of developing an adverse event during therapy is currently available. A multidisciplinary approach addressing appropriate interventions aimed at reducing the risk of any insidious, severe, and/or dose-limiting toxicity might represent the most efficacious strategy to timely prevent and manage severe irAEs, allowing indirectly to improve both patients’ cancer-specific survival and quality of life. In this review, we reported a five-case series of toxicity events that occurred at our center during treatment for mRCC followed by the remarks of physicians from different specialties, pinpointing the relevant role of an integrated and extended multidisciplinary team in a modern model of mRCC patient management.

## 1 Introduction

Renal cell carcinoma (RCC) is an insidious neoplasm, accounting for approximately 2% of global cancer diagnoses and deaths, whose incidence will further increase worldwide. Cancers of the kidney and renal pelvis have rapidly become more common in the developed world over the past decades (1). According to 2018 GLOBOCAN data, an estimated 403,000 people per year are diagnosed with kidney neoplasms, constituting 2.2% of all cancer diagnoses (2). In Italy, AIOM estimates that for the year 2020, the number of new cases of kidney cancer is 13,500 and deaths 4,900, accounting for 2.4% of all cancer-related deaths (3). The overall survival (OS) of patients affected by RCC has improved year after year: compared with the 90s and 2000s, an increase in OS has been shown, respectively, of 25% and 11%, both in USA and Italy, representing one of the best results obtained during the last 10 years (4). Indeed, with the arrival of new innovative molecules, such as immune checkpoint inhibitors (ICIs) and novel tyrosine kinase inhibitors (TKIs), the prognosis of RCC in advanced stages has been profoundly improved. According to European guidelines (5), the first-line treatment of metastatic RCC (mRCC) depends on the IMDC (International Metastatic RCC Database Consortium) risk group, defined by six negative clinical prognostic factors that stratify patients with mRCC in three subgroups: good, intermediate, and poor-risk. Accordingly, patients without negative factors have a good prognosis and may obtain a longer survival; patients with one or two factors are at an intermediate risk of death, with a median OS of about 23 months; patients with three or more factors are expected to have a poor outcome, with a median survival of about 8 months (6). The first-line therapy in the favorable-risk mRCC should be a TKI in combination or not with an ICI (in Italy, the current approved combination is axitinib plus pembrolizumab according to the KEYNOTE-426 trial (7); in the intermediate or poor risk, other than a TKI+ICI combination, dual immuno (IO) combination (IO–IO) with ipilimumab and nivolumab can also be used, according to the CheckMate 214 trial (8). Other combinations like cabozantinib and nivolumab, and lenvatinib plus pembrolizumab, as reported in the CheckMate 9ER (9) and CLEAR (10) studies, respectively, were recently approved by EMA for any IMDC risk class mRCC, but they are still not approved in Italy by AIFA; thus, we will not further discuss their use.

In KEYNOTE 426, the most common related grade 3 or higher adverse effects described are (≥10% patients in either group) as follows: hypertension [95 (22%) of 429 patients in the pembrolizumab plus axitinib group vs. 84 (20%) of 425 patients in the sunitinib group), alanine aminotransferase increase [54 (13%) vs. 11 (3%)], and diarrhea [46 (11%) vs. 23 (5%)]; deaths from adverse events (AEs) occurred in 19 (4%) of 429 patients in the pembrolizumab plus axitinib group (acute coronary syndrome, acute myocardial infarction, cardiac failure, cardiac tamponade, myocarditis, unknown cause, general physical health deterioration, sudden cardiac death, necrotizing fasciitis, pneumonia, plasma cell myeloma, myasthenia gravis, pleural effusion, pneumonitis, pulmonary embolism, pulmonary thrombosis, and respiratory failure, in one patient each; and cardiac arrest in two patients) (6). In CheckMate 214, the most common adverse reactions (≥20%) of any grade reported in patients treated with nivolumab plus ipilimumab (n = 547) were fatigue (58%), rash (39%), diarrhea (38%), musculoskeletal pain (37%), pruritus (33%), nausea (30%), cough (28%), pyrexia (25%), arthralgia (23%), decreased appetite (21%), dyspnea (20%), and vomiting (20%). The most frequent serious adverse reactions reported in ≥2% of patients were diarrhea, pyrexia, pneumonia, pneumonitis, hypophysitis, acute kidney injury, dyspnea, adrenal insufficiency, and colitis. Severe or fatal cases have also been reported with adverse reactions involving different organs and systems, especially cardiovascular (myocarditis, pericarditis, vasculitis), gastrointestinal (pancreatitis to include increases in serum amylase and lipase levels, gastritis, duodenitis), musculoskeletal and connective tissue (myositis/polymyositis, rhabdomyolysis, and associated sequelae including renal failure, arthritis, polymyalgia rheumatica), and endocrinological (hypoparathyroidism) diseases (8).

In case of disease progression, the most frequently used second-line treatment is the multi-tyrosine kinase inhibitor cabozantinib. However, a well-defined treatment algorithm has not yet been established (11). During cabozantinib treatment, most adverse reactions occur early in the course of treatment and include hypocalcemia, hypokalemia, thrombocytopenia, hypertension, palmar–plantar erythrodysesthesia syndrome (PPES), proteinuria, and gastrointestinal (GI) events (abdominal pain, mucosal inflammation, constipation, diarrhea, vomiting). In the METEOR trial, patients pretreated with vascular endothelial growth factor (VEGF)-targeted therapy reported dose reductions and dose interruptions due to an AE in 59.8% and 70%, respectively. Finally, when cabozantinib was given in combination with nivolumab in first-line advanced renal cell carcinoma, according to the most recent trial, CheckMate 9ER, dose reduction and dose interruption of cabozantinib due to an AE occurred in 54.1% and 73.4% of patients. The rates of treatment-related adverse events of grade 3 or higher were 60.6% (6.9% diarrhea, 7.5% PPES, 12.5% hypertension, 5.3% increased ALT level, 9.4% hyponatremia, 5.9% hypophosphatemia) in the nivolumab-plus-cabozantinib group and 50.9% (4.4% diarrhea, 7.5% PPES, 13.1% hypertension, 4.7% decreased platelet count and 3.8% neutropenia/anemia) in the sunitinib group.

New targeted agents as well as a new combo with immunological drugs expand treatment chances for mRCC patients but are associated with more novel toxicities as compared with those observed with the previously available medications, such as sunitinib or pazopanib. Moreover, due to the relatively recent introduction of these combinations in clinical practice, their cumulative dose adverse effects are still unknown. However, the most frequently occurring affect the skin, colon, endocrine organs, liver, and lungs. Others are very infrequent but may be very serious, even lethal, such as neurological disorders and myocarditis (12).

A prompt recognition and management of these toxicities represents a fundamental issue in oncological clinical practice, since it correlates with the outcome of cancer patients. In this context, it is therefore essential to prevent any adverse events that may lead to a discontinuation of treatment or a dose reduction. A multidisciplinary management of the various toxicities that may arise during treatment of patients with mRCC will obviously help patients to achieve better treatment compliance (10). Indeed, multidisciplinary teams (MDTs) have been recommended to improve cancer care and outcomes for all managed patients (13). Patients should be investigated for preexisting risk factors to contain the effect of those that are modifiable, even if consensus recommendations for the identification of a population most at risk of toxic events are currently lacking. For those patients with baseline organ impairments, a multidisciplinary approach should be strongly recommended for an early identification of potential adverse events. The limited knowledge of the pathophysiology and management of life-threatening complications relating to new cancer drugs presents a need to provide a more heterogenous staff, with oncologists, and organ specialists with evidence-based algorithms and requires a multidisciplinary approach (14).

Nowadays, there is no specific protocol to evaluate the risk of developing an adverse event from the novel therapies for mRCC patients. Therefore, we reported a case series and literature review, describing five examples of critical toxicities that occurred in our center during treatment for mRCC and how they should be managed, with the aim to highlight the role of MDT in the genitourinary cancer unit for an integrative management of mRCC patients.

## 2 Patients and method

This study reported a case series of mRCC treated at Sapienza University Oncological Units with a special focus on the different toxicities that occurred during IO-based or targeted therapies for mRCC. Clinical records of five patients affected with clear cell renal carcinoma, treated in metastatic setting, and discussed in our multidisciplinary team for drug-related toxicity were analyzed for the present study. The first case reported a multidisciplinary management of endocrinological toxicity during the IO combo with nivolumab (3 mg per kilogram of body weight) plus ipilimumab (1 mg per kilogram) intravenously every 3 weeks for four doses, followed by nivolumab (3 mg per kilogram) every 2 weeks. The second case involved a patient, treated before with pembrolizumab plus axitinib at a standard schedule (pe 200 mg plus axi 5 mg twice a day, administered at a 3-week interval) followed in second line with cabozantinib, who reported nephrological toxicities. The third and fourth cases entailed patients treated in the first-line treatment with standard pembrolizumab plus axitinib, during which they showed liver and cardiological toxicities, respectively. Finally, the fifth case was about a multidisciplinary management of dermatological toxicity due to cabozantinib. The severity of adverse events was graded according to CTCAE version 4.0. At the time of first oncological visit, all our patients signed informed consent in which the consent to the use of their data for research purposes is included.

## 3 Results

### 3.1 Case 1: Multidisciplinary management of endocrinological toxicities

#### 3.1.1 Case presentation

**Figure 1:**
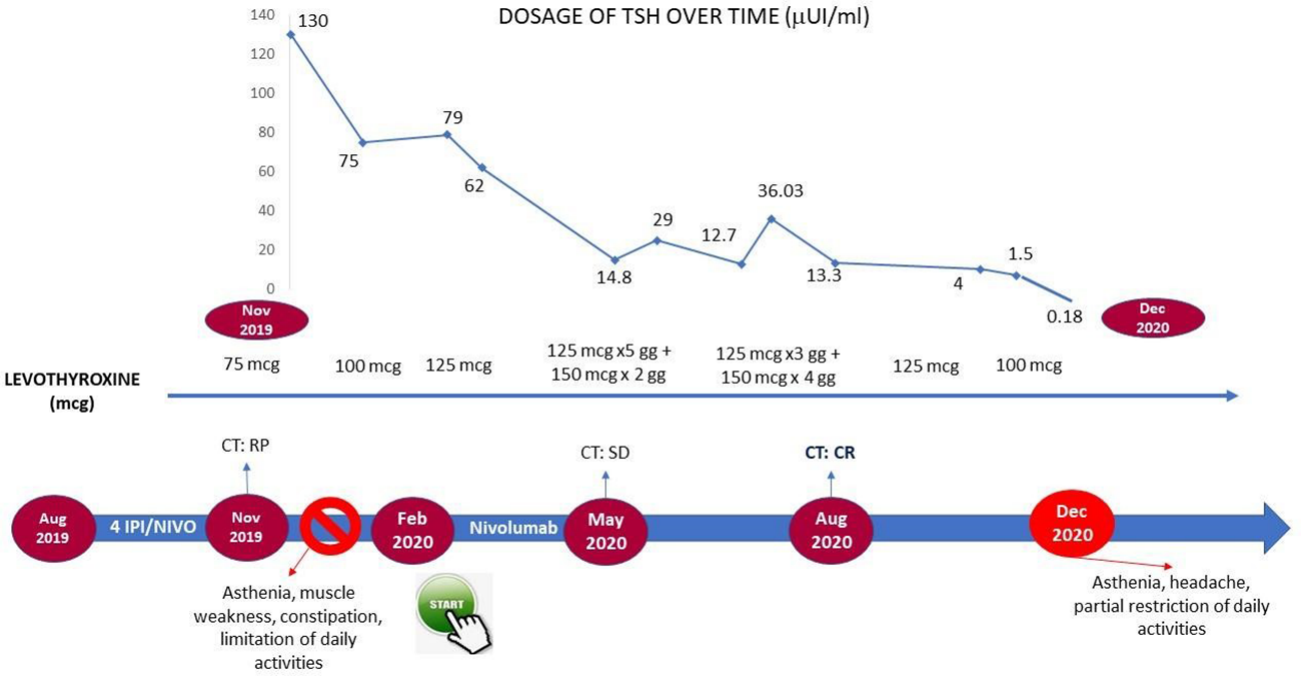
Timeline management of cutaneous toxicity in the course of cabozantinib treatment.

A 69-year-old man underwent right nephrectomy surgery in May 2019 for a renal carcinoma with sarcomatoid (Ki67 40%, p53 <1%) and poorly differentiated clear renal cell components, pT3a pNx, stage III according to AJCC 2017. The postsurgery total-body contrast-enhanced computed tomography (CT) showed suspected pulmonary and mediastinal lymph node metastasis, confirmed by transbronchial needle aspiration (TBNA). According to the prognostic criteria of Motzer and Coll and Heng (15, 16), for the presence of hypercalcemia and the time to start systemic treatment less than 1 year after diagnosis, the patient belonged to the intermediate prognosis group. In August 2019, he began immunotherapy with nivolumab (3 mg per kilogram of body weight) plus ipilimumab (1 mg per kilogram) intravenously every 3 weeks for four doses, followed by nivolumab (3 mg per kilogram) every 2 weeks. In view of the combination of an anti-CTLA4 and an anti-PD1, a periodic monitoring of thyroid function (TSH, FT3, FT4), for each of the first four doses, and hypophyseal function (basal ACTH and cortisol) was performed (17). At the third administration, we observed a grade 1 (G1) hyperthyroidism according to Common Terminology Criteria for Adverse Events 5.0 (CTCAE) [↓TSH 0.03 µIU/ml (normal range 0.27–4.2), ↑FT4 2.29 ng/dl (normal range 0.7–1.48), FT3 2.5 pg/ml (normal range 1.71–3.71)], without related symptoms, and as recommended by guidelines, immunotherapy was continued with laboratory monitoring. At the fourth cycle, the G1 hyperthyroidism was stable. The revaluation CT showed a partial response and nivolumab was continued. At the first maintenance cycle, the patient was asthenic, with muscle weakness, constipation, and limitation of daily activities. Laboratory tests showed normal pituitary function and confirmed G2 hypothyroidism [TSH 130.0 µIU/ml (0.27–4), ↓ FT4 0.10 ng/dl (0.7–1.48), FT3 2.0 pg/ml (1.71–3.71) ↑ thyroglobulin 187 ng/ml (normal range 3–40)]. Treatment was discontinued until control of symptoms, from December 2019 to February 2020, and a different thyroid hormone supplementation with levothyroxine was prescribed (**Figure 1**). In February 2020, the patient started therapy with nivolumab, reaching in August 2020 an optimal response with a resolution of hypothyroidism at the end of October 2020.

**Figure 2:**
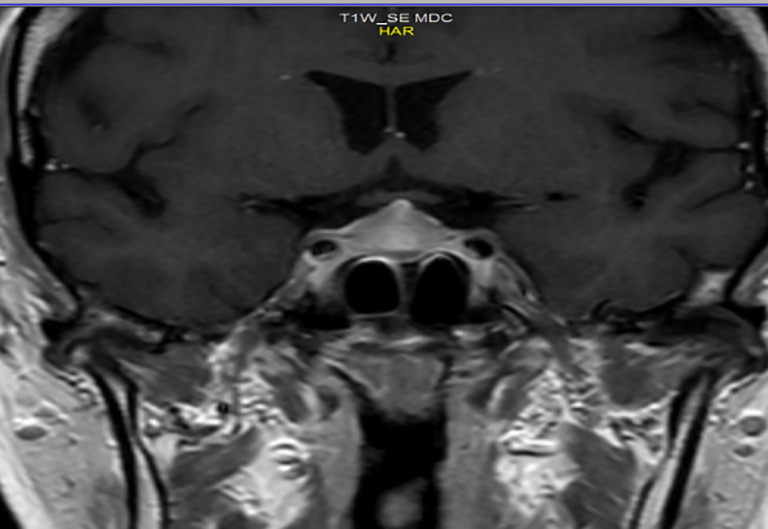
MRI: Radiological signs of hypophysitis. Inhomogeneous and enlarged appearance of the pituitary gland showing “tent” morphology due to tension of the meninges with thickening of the pituitary stalk.

In December 2020, the patient had G2 asthenia, restriction of activities of daily living but not of personal care, dizziness, headache, non-alterations of vision, and G1 diarrhea. Laboratory tests showed hypoglycemia (72 mg/dl), hyponatremia, reduced levels of ACTH (5.4 pg/ml; normal range 7.2–63.3 pg/ml), and cortisol (3.5 µg/l; normal range 23–194 µg/l) at 8:00 a.m., TSH (0.18 µUI/ml), and FT4 (0.6 ng/dl). The ACTH stimulation test (1 µg) showed an insufficient adrenal response (basal cortisol: 3.3 µg/dl; cortisol 60 min: 6.8 ug/dl). Due to the headache and dizziness, a magnetic resonance imaging (MRI) of the brain was performed and highlighted the radiological signs of a meningeal irritation attributable to an hypophysitis (**Figure 2**).

Then, in the presence of secondary adrenal insufficiency and secondary hypothyroidism, a diagnosis of immuno-related hypophysitis was placed, also supported by radiological imaging. The ICI was stopped, and an adrenal and thyroid replacement therapy (levothyroxine, 125 µg in the morning and cortisone acetate 25 mg upon awakening and 12.5 mg in the early afternoon) was administered. The immunotherapy was suspended for a month and resumed after normalization of pituitary function. The patient still maintains a complete radiological response with an OS of 29 months.

#### 3.1.2 Endocrinologist opinion

The incidence of thyroid disorders in course of immunotherapy is rarely higher than G2, due to the frequent monitoring of thyroid function that allows to an early detection. In particular, a meta-analysis of 28 studies, which included more than 7,500 patients, showed an incidence of hyperthyroidism and hypothyroidism, under combined anti-CTLA4/anti-PD1 treatment, of 8% and 13.2% versus 3.2% and 3.9% in the course of an anti-PD1 treatment, respectively (8). Thyroid disorders are more frequently primary, rarely secondary to pituitary gland dysfunction. Both the hyper- and hypothyroidism are different manifestations of the same pathological entity: a dextrose thyroiditis mediated by cytotoxic T lymphocytes against the thyroid gland (9, 10). Nowadays, international guidelines do not provide a clear direction regarding the management of G2 hypothyroidism. In fact, according to the AIOM Italian guidelines (11), ICI treatment should be continued, associating it with hormone replacement therapy, whereas the ESMO and ASCO guidelines give the opportunity to stop treatment according to clinical judgment (17, 18). Although many of the studies in the literature are retrospective, in most cases the immunotherapy is continued without further toxicity (19–26). In the clinical case described, on the contrary, the treatment was discontinued for about 3 months.

Pituitary gland disorders are more frequent with anti CTLA-4 than with anti-PD1/PD-L1. The incidence of hypophysitis depends on the dose and drug administered: with ipilimumab, 3 mg/kg is 1%; with ipilimumab, 10 mg/kg is 16%; with nivolumab, 240 mg is 1.1%; and with ipilimumab, 3 mg/kg + nivolumab 240 mg reaches 8% (8, 20). The pituitary damage is apparently caused by monoclonal antibodies and/or activation of T cells directed against antigens shared between cancer cells and pituitary cells or cross-reactive antigens (17, 27, 28). Currently, the guidelines recommend discontinuing treatment and setting up an endocrine replacement therapy, in case of G≥2 immuno-related hypophysitis (17, 18). However, even in this case, in several retrospective studies some patients, despite a G2 toxicity, continued immunotherapy with good control of symptoms (24, 29, 30). It is our opinion to assess the possible interruption on a case-by-case basis, discussing the choice in a multidisciplinary team, as in some circumstances, a good control of symptomatology can be obtained without interrupting the ICI treatment ongoing.

In conclusion, in the present case report, the front-line treatment, still in progress, has been allowed to reach a survival of more than 2 years. An adequate laboratory monitoring is mandatory to manage endocrine toxicities in advance. Of course, a more appropriate diagnostic classification of endocrinological toxicity, together with a more detailed of toxicity degree, is required. In case of G≥2 immuno-related endocrinological disorders, suspension of treatment is not mandatory. A multidisciplinary approach in the management of toxicity is essential to ensuring a correct cost/benefit balance for the patient, favoring therefore greater adherence to treatment while respecting an adequate quality of life. **Table 1** shows the biochemical tests that should be performed during treatment with ICIs.

**Table 1:** The table summarizes our MDT suggestions and does not reflect any expert consensus or guideline: which exams are recommended by the experts to prevent and identify any adverse event?.

Category of toxicity	Which exams are recommended?	When and How?
Endocrinological	ACTH, baseline cortisol, TSH, FT3, FT4	For anti CTLA4 (alone or in combination): every cycle for the first 4 cycles then every 4-6 week
		For anti PD1 or anti PD-L1: every cycle for first 3 months and every second cycle thereafter (cortisol is indicated by symptoms/falling TSH)
		When morning cortisol values are between 3 and 15 ug/dl.
	ACTH test	Peak cortisol levels <18.1 ug/dl at 60 minutes indicates adrenal insufficiency.
Nephrological	Renal function, urine analysis including 24 hours proteinuria and electrolytes	Baseline and every cycle
Liver	Hepatitis baseline screening	Baseline
	Liver function test	At the first occurrence of liver enzyme increase
		Every cycle
Cardiovascular	Blood pressure measurement;	Baseline and weekly in the first 8 weeks
	Comprehensive cardiological evaluation including electrocardiogram, troponine and NT-pro BNP, echocardiogram with strain analysis;	Baseline and in case of symptoms
	Cardiovascular Magnetic Resonance;	In case of symptoms and/or troponine raise and/or ECG change
Dermatological	Clinical examination	Every cycle
	Dermatological evaluation	At baseline in case of patients with history of skin disease At symptoms
	RF dosage and HLA genotype testing	Baseline (only within clinical trial)

In summary, our case concluded that adequate laboratory monitoring is essential for early intervention in the management of endocrine toxicity. In the presence of endocrinopathy, an accurate diagnosis and a correct definition of the degree of toxicity are needed; if hypothyroidism and adrenal insufficiency occur during treatment with ICIs, it is extremely important to start replacement therapy but discontinuation of immunotherapy is almost never indicated. A multidisciplinary approach to the management of toxicities is essential to ensuring correct continuation of therapy for the patient and also greater adherence to treatment in accordance with an adequate quality of life.

### 3.2 Case 2: Multidisciplinary management of nephrological toxicities

#### 3.2.1 Case presentation

In June 2008, a 70-year-old man with a history of cerebral ischemia, atrial fibrillation, and hypertension experienced a persistent abdominal pain and weight loss (5 kg over a year). Imaging revealed a renal lesion of 58 × 62 × 55 mm in the upper pole and pars intermedia of the right kidney, suspected for neoplastic mass, with no other tumor lesions. Therefore, the patient underwent right nephrectomy, and the histological examination revealed a clear-cell type cancer (pT1b pN0, stage I according to TNM/AJCC classification and G2 according to Fuhrman classification). The follow-up was negative until a total body CT scan showed a relapse of disease in the lung, pancreas, and subcutaneous tissue (the histological examination revealed a new metastatic lesion of clear-cell type carcinoma). In January 2021, a first-line therapy for good risk with standard pembrolizumab plus axitinib was administered. Laboratory tests documented a baseline serum creatinine at 1.4 mg/dl. After 5 cycles, in May 2021, for the first time the renal function worsened (serum creatinine 2.1 mg/dl) with a negative urine test. Renal ultrasound did not show any sign of kidney obstruction (e.g., calculi deposits). The patient carried out a nephrological evaluation, and it was decided to replace sartan-based antihypertensive therapy with a calcium antagonist in order to avoid concomitant renal medication damage. Hydration was preserved, and cancer treatment continued. After 2 weeks, creatinine was about 1.5 mg/dl. Oncologic therapy was not stopped. After 2 more cycles of pembrolizumab plus axitinib, acute renal dysfunction was observed again (serum creatinine 2.4 mg/dl). Therefore, according to the ESMO clinical guidelines about the management of immunotherapy-related nephritis (4) and in agreement between oncology and nephrology specialists, therapy with pembrolizumab plus axitinib was withheld, a correct state of hydration was guaranteed, and prednisolone 0.5 mg/kg/die was started. After 2 weeks, serum creatinine was about 1.8 mg/dl and we decided to restart axitinib. After 2 more weeks, serum creatinine was 1.5 mg/dl and combined therapy with pembrolizumab and axitinib was resumed, continuing prednisone 5 mg per day (31).

In September 2021, after nine cycles of therapy, a total body CT showed disease progression: the pancreatic nodule increased in size (from 1.5 × 1 cm to 3.5 × 5.5 cm) and a new lesion appeared in the second liver segment. Thus, we decided for a second-line treatment with cabozantinib 60 mg per day. After 3 cycles, in November, hypertension had worsened and there was a gradual, progressive deterioration of renal function: creatinine was about 1.95 mg/dl and urinalysis revealed proteinuria = 50 mg/dl and microhematuria. The daily urine protein loss was found to be about 1,200 mg. Hydration was started and cabozantinib 60 mg per day continued. After 2 weeks, serum creatinine was still about 1.85 mg/dl and daily urine protein loss was 1,000 mg. Then, in agreement between oncology and nephrology specialists, cabozantinib was reduced to 40 mg per day. After 2 weeks, serum creatinine was 1.7 mg/dl, daily urine protein loss was 600 mg, and after 2 more weeks creatinine was 1.6 mg/dl and daily 24-h urine protein loss was 250 mg.

**Figure 3:**
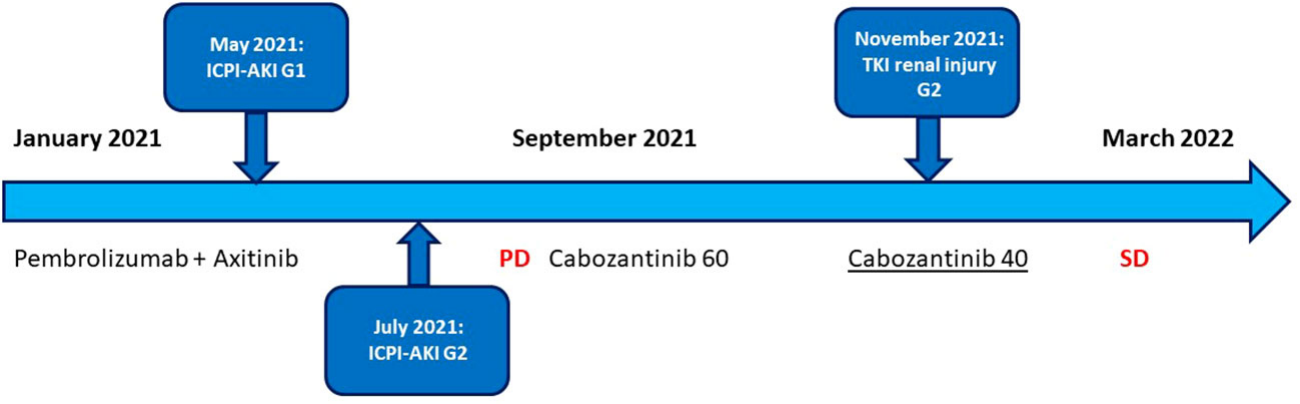
Timeline management of nephrological toxicity in course of ICI- and TKI-based treatment.

Then, the patient continued cabozantinib 40 mg per day, no more renal toxicity was observed, and the treatment was well-tolerated. In March 2022, total body CT showed stable disease and treatment with cabozantinib 40 mg per day is still ongoing (**Figure 3**).

#### 3.2.2 Nephrologist opinion

According to the advent of new oncological treatments (for example, combinations of immunotherapy–immunotherapy and TKI–immunotherapy), drug toxicity and especially renal toxicity are more frequent than before. As reported by ESMO clinical guidelines (17), for example, renal dysfunction is rare with ipilimumab and with anti-PD-1 therapies, described in <1% of treated patients (32). The incidence is higher with combination of ipilimumab plus nivolumab, reaching 4.9%, with 1.7% of grade 3 to 4 toxicity. Similarly, sequential therapy with ipilimumab followed by nivolumab is associated with a high incidence of 5.1% (33).

In order to manage different types of toxicity, a timely treatment of renal injury is crucial and in this setting the nephrologist’s role is of primary importance inside the MDT.

Furthermore, a new evolving field, namely, onco-nephrology, has emerged during the last few years. It includes the vast spectrum of renal disorders that can arise in patients with cancer. A differential diagnosis between progression of underlying renal disorders and secondary disorders due to oncological treatments or to the malignancy itself is essential in order to allow the oncologist to continue the antineoplastic therapy. Cancer therapy is increasingly prescribed in elderly patients, a population often already affected by multiple morbidities and preexistent CKD (chronic kidney disease). Therefore, it is important to consider how the presence of CKD, AKI (acute kidney injury), and other renal disorders may affect treatment options and outcome and how certain therapies may increase the risk of kidney toxicity (34).

Regarding our case report, it is well known that VEGFR2 inhibitors lead to some adverse events such as proteinuria, hypertension, hand−foot syndrome, and kidney dysfunction whereas ICIs lead to other adverse events as autoimmune disorders, such as thyroiditis, colitis, skin disease, and different forms of nephritides.

Clinically, renal adverse effects of anti-VEGF therapies are arterial hypertension, proteinuria, rarely nephrotic syndrome, AKI, or CKD. Various parts of the nephron can be injured; 42% of the total number of renal adverse effects is represented by renal impairment, 47% by metabolic disturbances, and hypertension in 11% (31).

Podocytes and endothelial cells are involved, resulting in severe alteration of the architecture and function of the GBM (35).

Proteinuria is described as one of the most common renal side effects of other anti-VEGF drugs and frequently occurs with hypertension (36). It is the result of glomerular filtration barrier impairment in the glomeruli, releasing an abnormal amount of plasma proteins, mainly albumin, in urine, and it is a direct marker of therapy nephrotoxicity. The incidence and rate of proteinuria are variable, and the incidence of all grades of proteinuria during the treatment with cabozantinib was about 12%, whereas no one was with severity > grade 3. Despite this high frequency, most cases of proteinuria are asymptomatic or not severe (37–39).

Proteinuria, hypertension, and kidney injury are closely related to the destruction of the integrity of the glomerular filtration barrier, composed of podocytes, a glomerular basement membrane, and endothelial cells. TKI-induced endothelial cell damage leads to the compensatory expression of pro-angiogenic factors and the formation of an abnormal endothelial−podocyte cross talk and podocyte injury (40).

On renal biopsy from patients receiving TKIs, the most common pathological findings are thrombotic microangiopathy (TMA), MCD (minimal change disease), and FSGS (focal segmental glomerulosclerosis), as the result of direct cellular toxicity on endothelial cells and/or podocytes (40–42). In addition, some drugs can cause damage to different tubular transporters. For example, according to submitted studies the incidences of hypopotassemia and hypomagnesaemia were about 11% and 16% during the treatment with cabozantinib (38, 43).

Despite TKIs, ICIs can cause different autoimmune diseases known as immune-related adverse events (irAEs) (44). Usually, kidneys are less involved; however, up to now, ICI-associated AKI (ICPI-AKI) has posed challenges in diagnosis and management (44–47). Renal histopathology mainly reveals ATIN (acute tubular–interstitial nephritis). Different causes of AKI should be considered, and these are also important to decide about treatment with steroids and/or interruptions of ICI therapy without leading to tumor spreading and/or irreversible organ damage.

A recent multicenter study identified three independent risk factors for development of ICI-AKI: 1) concomitant use of PPIs; 2) combination treatment with anti–CTLA-4 and anti–PD-1/PD-L1 agents; and 3) lower baseline eGFR (48).

According to the results of this study, patients receiving PPIs, those receiving combination ICPI therapy, and those with a lower baseline eGFR may receive closer renal surveillance.

In this setting, the figure of the onco-nephrologist is very important for the consultation and consideration of kidney biopsy, especially for patients with persistent stage 1 AKI, and those who develop stage 2 or 3 AKI.

Indeed, in a recent review (49), patients who develop stage 1 AKI treated empirically with steroids whose kidney function does not improve should undergo kidney biopsy to assess for alternative etiologies of AKI (e.g., glomerulonephritis, which may require additional immunosuppressive therapies). Patients with stage 2 or 3 AKI who have plausible alternative etiologies for AKI other than ICIs should proceed directly to kidney biopsy.

Kidney dysfunction under TKIs usually resolves with dose reduction or drug discontinuation, and it depends, in part, on the patient’s baseline serum creatinine. However, these patients also present many risk factors for CKD such as diabetes, old age, hypertension, and nephrectomy which can lead to chronic kidney failure. In the KDIGO Controversies Conference on onco-nephrology, for patients with CKD, TKIs may be used at a lower-than-standard dose and then increased according to individual tolerability (50). According to international clinical guideline recommendations for the management of immune-related adverse events, including ICI-AKI, currently, there are no therapies to treat these renal complications, apart from drug discontinuation, dose reduction, or symptomatic treatment. Thus, this is really important in order to better understand the underlying mechanisms to reduce nephrotoxicity without inhibiting the anti-angiogenic effects on cancer.

Recommendations for management of ICI-associated adverse renal effect have been recently summarized in a complete review by Hermann and Perazella (51).

International guidelines suggest the following management of immune-related adverse events (15, 50, 51): in case of ICPI-AKI grade G1, the treatment with ICI can be continued; in case of ICPI-AKI grade 2, the treatment with ICPI should be suspended and restarted once serum creatinine is back to grade G1. For patients with ICPI-AKI grade 2 or more, such as the ICPI-AKI described in this case report, steroid treatment may start with prednisone 0.5–1 mg/kg/day for G2, 1–2 mg/kg/day for refractory G2 and for G3–G4.

ASCO guidelines also suggest the use of other immunosuppressive agents like mycophenolate mofetil, azathioprine, cyclophosphamide, and infliximab if corticosteroid therapy is not enough (52).

Nevertheless, in Italy these immunosuppressive agents are not recommended in immune-related adverse events and so we cannot express an opinion on this subject.

However, according to these findings, baseline renal function, urine analysis, and electrolytes are three of the most important things to monitor during cancer treatment with both TKIs and ICIs, especially in patients with comorbidities (diabetes, arterial hypertension) that can cause one of the most difficult problems for making the differential diagnosis between collateral effects of antineoplastic drugs or preexistent diseases. Therefore, a complete evaluation of kidney function prior to oncological therapies is mandatory for prolonging the survival of our patients (53) (**Table 1**).

### 3.3 Case 3: Multidisciplinary management of liver toxicity

#### 3.3.1 Case presentation

**Figure 4:**
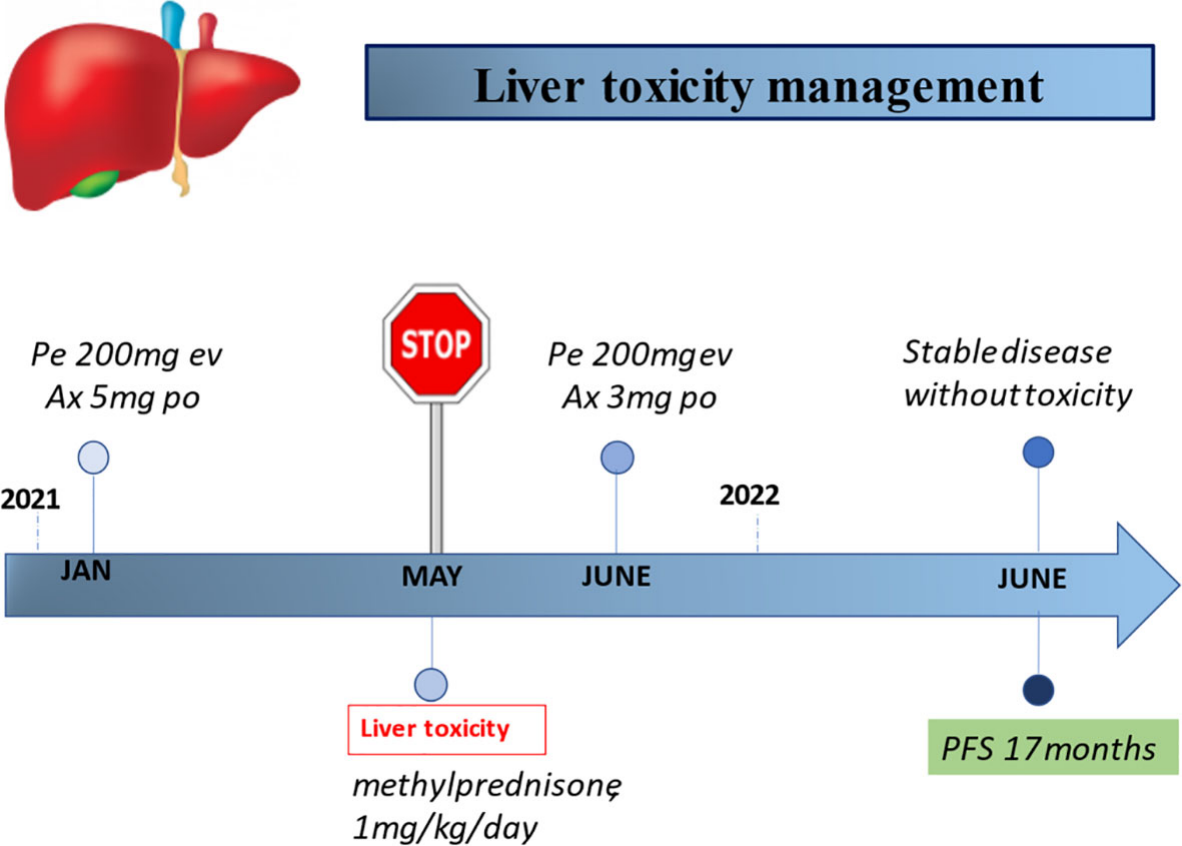
Timeline of the management of liver toxicity during ICI-based treatment. Pe, pembrolizumab; Ax, axitinib.

In September 2019, a 63-year-old woman, with a past medical history of active smoking (two packs a day for the last 15 years), who experienced a progressive weight loss and dyspepsia, underwent abdominal ultrasound and CT scan which showed a huge expansive mass (10.5 × 7.8 cm) at the level of the middle and lower portions of the left kidney. She thus underwent left nephrectomy. Histopathological report diagnosed a clear cell-type RCC, grade 2 Fuhrman nuclear grading, pT2b pN0 according the AJCC TNM system. Follow-up was negative until June 2020, when a chest CT scan showed at the level of the left lung the increase of both a parascissural nodule, measuring 10 × 9 mm (previously 2.5 mm), and of a nodule in the posterobasal segment (measured 4.5 vs. 3.5 mm). She underwent atypical pulmonary resection, and the histological examination described a pulmonary localization of RCC. After 2 months, due to acute dyspnea, she underwent a further CT scan which showed left lung pleural effusion and necrotic solid tissue localized at the apex of the left lung (10 × 3.3 cm), which was infiltrating the pleura, pericardium, and fifth rib; additional pleural implants; carcinomatous lymph nodes; and mediastinal lymphadenopathies. The patient then had blood tests which showed levels within a normal range. According to the IMDC, for Karnofsky Performance Status below 80%, she was categorized prognostically as at an intermediate risk. Consequently, from January to May 2021, she underwent administration of six cycles of pembrolizumab + axitinib with partial radiological response, and her global clinical conditions improved. In May 2021, after the sixth cycle, routine biochemical tests showed an increase in serum transaminases, with a normal bilirubin value: the glutamic-oxaloacetic transaminase (GOT) value was 83 U/l (normal value ≤32 U/l) whereas the glutamic-pyruvate transaminase (GPT) was 142 U/l (normal value ≤33 UI/l). According to the classification NCI-CTCAE (v.5.0) (54), the patient had a grade 2 liver toxicity. As soon as the increase in transaminases was detected, a hepatological consultation was requested. Other potential causes of liver toxicity were ruled out (e.g., viral, autoimmune, alcohol, use of medications, supplements, or herbal products); no other alteration of liver tests (e.g., total bilirubin, alkaline phosphatase, gamma glutamyl transferase (GGT), coagulation tests, electrophoretic protidogram) was detected. The patient did not report any abdominal complaint, and physical examination did not show either hepato- or splenomegaly. Ultrasound did not detect liver metastases. According to the analyzed values (**Table 1**), an immune-related liver toxicity was diagnosed. Both drugs were stopped, and according to the current guidelines, oral steroids (prednisone, 1 mg/kg/day) were started. Follow-up biochemistry performed after 2 weeks of steroid treatment showed normal liver tests (GOT 16 U/l; GPT 30 U/l), and prednisone was then progressively tapered and stopped. Thanks to the help of the hepatologist and medical therapy, liver toxicity quickly resolved, and the patient resumed the scheduled treatment with pembrolizumab and axitinib at a reduced dosage of 3 mg daily bid, which is currently ongoing with good compliance and clinical results (**Figure 4**).

#### 3.3.2 Hepatologist opinion

During ICI monotherapies (such as ipilimumab, nivolumab, and pembrolizumab), liver enzymes’ increases in various orders of magnitude have been reported to cardiological toxicityoccur in around 2%–10% of patients (1). In case of combination treatments, these increases tend to occur more frequently, with figures as high as 25%–30% of all grade toxicities in the case of ipilimumab and nivolumab, whereas incidence of G3 toxicity occurrence is more limited, at an approximate 15% incidence rate (52, 55, 56). Anti–PD-1/PD-L1 seems to have a lesser incidence of liver-related IrAEs of any grade as compared with anti–CTLA-4 (1, 3). Liver failure with encephalopathy in the context of acute fulminant hepatitis remains instead a rare evidence, occurring in 0.4%–0.14% of treated patients (56). The onset of liver enzyme alterations usually develops within the first 6–12 weeks after treatment initiation (57); even with some discordances, some authors have suggested different timings of onset for the different ICIs (anti-CTLA-4 vs. anti-PD-1 vs. anti-PD-L1) (17, 56) and an earlier occurrence of adverse events in case of combination treatments as compared with monotherapies (56). Data on ICI retreatment after an episode of drug-related liver adverse events are very poor. In a large retrospective study, among patients who had resumed ICI treatment after transaminase decrease after temporary drug discontinuation, 26% of them developed recurrence of hepatotoxicity (58).

Liver-related adverse events (LRAEs) occurring during ICI treatment are usually reported and scored according to the CTCAE (54). It has been recently suggested that the preferred term to denominate cases of liver injury caused by ICIs should be “immune-mediated liver injury caused by immune checkpoint inhibitors” (ILICI) (56). However, in a more hepatological perspective, we suggest to first define the pattern of liver enzyme elevation by calculating the ratio of serum alanine amino transferase (ALT) to alkaline phosphatase (ALP) levels (R value = [ALT/upper limit of normal (ULN)]/[ALP/ULN]), which allows to categorize the event as hepatocellular (R >5), mixed (R >2 to <5), or cholestatic (R <2). Different R patterns may help in characterizing the observed adverse event and help to distinguish which drug is more probably involved in determining it. Nevertheless, it should be underlined that LRAEs are only imperfectly described by defining the increase in either hepatocellular or cholestatic indexes. Evaluation of liver damage severity must also include check of liver synthetic function, as expressed by laboratory parameters such as coagulation (prothrombin time/international normalized ratio), and total serum bilirubin, and by the presence of hepatic encephalopathy/ascites on the bases of, respectively, clinical and ultrasound findings; in fact, acute hepatitis is considered severe if the INR is >1.5, bilirubin is elevated (usually >2X ULN), and fulminant (i.e., potentially leading to hepatic failure), if impaired coagulation is accompanied by hepatic encephalopathy and/or prolonged jaundice and/or onset of ascites (59).

At present, liver biopsy should be considered for patients with more severe liver toxicity (grade >3) or in case of uncertain diagnoses. Patterns of liver toxicity during ICI treatment are still currently scarcely characterized from a histological standpoint (56), and biopsy of the liver might be useful to optimize the management in elusive cases of persistent/refractory LRAE, if blood tests or imaging evaluation does not provide conclusive information. In addition, to avoid misnomer, it has been pointed out that until a larger histological database of patients with suspected ILICI will be available, the term “hepatitis” should only be reserved for patients who have histological findings consistent with this entity. Also, in case of a prevalent cholestatic serum pattern, terms such as “cholangitis” should be avoided and only reserved for those who have either supportive histological findings or results of other reliable diagnostic tests (59).

Interestingly, ICI-induced liver toxicities do not display the histological (e.g., lack of plasma cells) and serological (absence of autoantibodies) features of an autoimmune hepatitis (AIH), but this notwithstanding, the current pharmacological management is mainly based on protocols derived from those used in the treatment of this liver disease (56). As recently reviewed (52), the mainstay of treatment is based on the use of either oral or intravenous steroids in various dosages and dose-escalation protocols, whereas the use of other immunosuppressant commonly employed in the management of AIH, such as oral mycophenolate mofetil, still needs further proof of efficacy and safety. In this setting, the use of infliximab is contraindicated for the concerns regarding its intrinsic hepatotoxicity. Furthermore, it should be underlined that liver transplant, as an option for the management of ICI-induced liver failure, is unfortunately not considered, since patients are affected by malignant tumors.

### 3.4 Case 4: Multidisciplinary management of cardiological toxicities

#### 3.4.1 Case presentation

A 74-year-old woman, due to persistent cough and abdominal pain on the left side, underwent an abdominal ultrasound and a total body CT scan that showed a mass of 2 cm in the left kidney, suspected for primary tumor, and multiple nodular lung lesions. After 1 month, in December 2020, the patient underwent a left radical nephrectomy, whose histological examination revealed a clear-cell type RCC (pT3a pNx according to the AJCC 2017 classification, 8th edition). After 2 months, the patient was referred to our center for the first oncological evaluation. The past medical history comprised systemic hypertension treated by angiotensin II receptor blockers and paroxysmal atrial fibrillation on direct oral anticoagulants (DOACs), allergic asthma on foster therapy, and type 2 diabetes mellitus treated by metformin. An echocardiogram showed a normal bi-ventricular dimension, wall thickness, and systolic function. The patient was in good general condition, the blood pressure was within the normal limit, the blood test showed normal renal function, and electrolytes were within limits, without proteinuria. According to the IMDC intermediate risk, a combination therapy with pembrolizumab and axitinib was started. The patient was instructed to monitor the blood pressure at home and to contact the clinic in case of hypertension or any new symptoms. After the third cycle of therapy, the patient reported asthenia and headache. The blood pressure was increased (180/90 mmHg). The patient was referred to a cardiologist. The electrocardiogram showed a sinus rhythm with a heart rate 90 bpm without repolarization changes. The blood pressure was persistently increased. Troponin showed a negative result. Echocardiogram showed a normal bi-ventricular systolic function with FEVS 61%. Antihypertensive therapy was implemented with the addition of amlodipine with good response to therapy. Furthermore, due to the drug interference between amlodipine and metformin by pharmacodynamic antagonism, the patient was closely observed for the risk of hypoglycemia.

**Figure 5:**
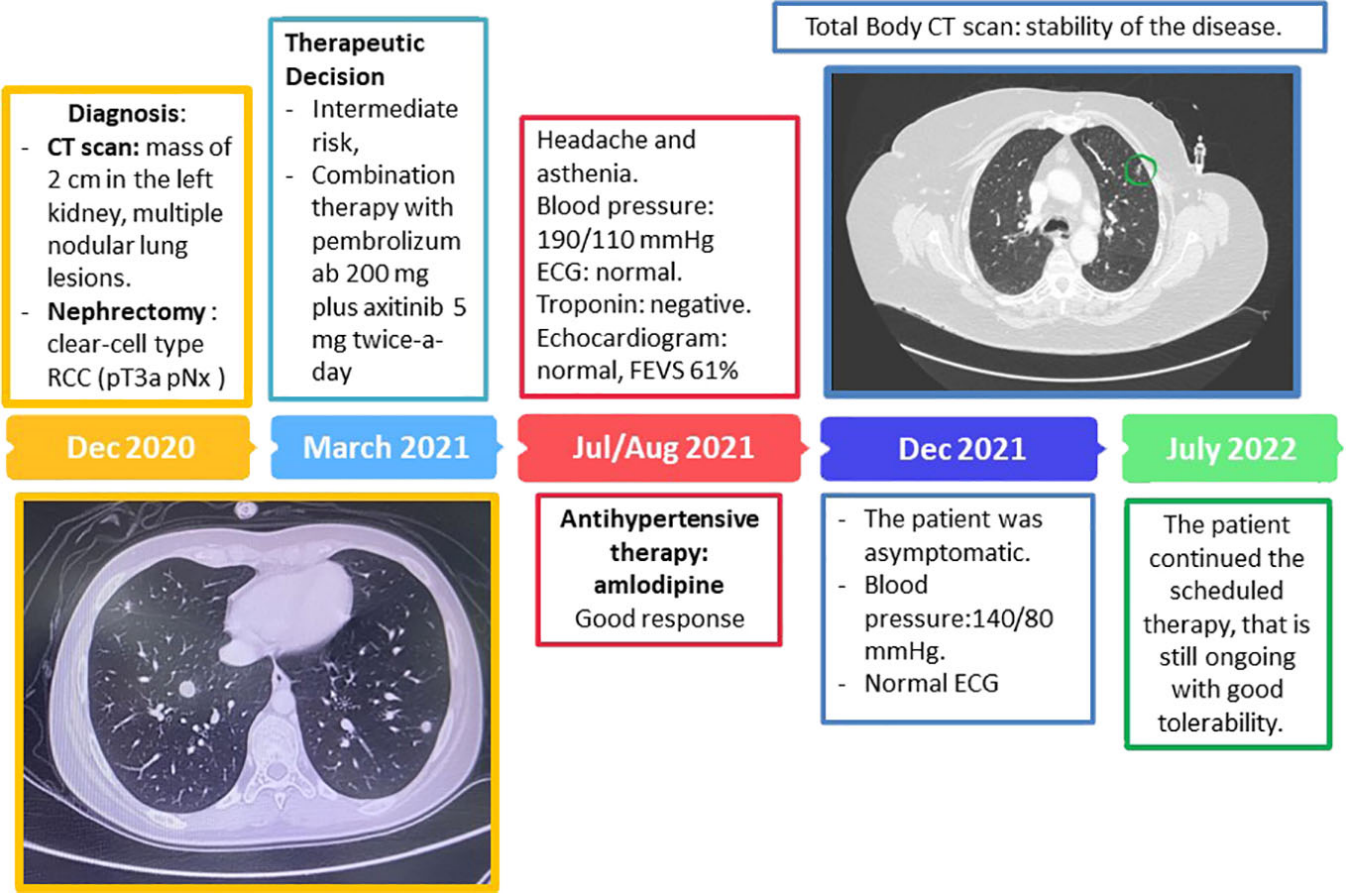
Timeline management of cutaneous toxicity in the course of cabozantinib treatment.

After 4 months, a total body CT scan was performed, showing stability of the disease. Blood test showed kidney function, electrolytes, and glucose levels within the normal limits, and no proteinuria. Cardiovascular evaluation showed normal ECG and normal blood pressure (140/80 mmHg). The patient was asymptomatic. Therefore, considering the stability of the disease, the results of the laboratory tests, the cardiovascular evaluation, and the improvement in symptoms, the patient continued the scheduled therapy, which is still ongoing with good tolerability (**Figure 5**).

#### 3.4.2 Cardiologist opinion

In this case, a combination of two different classes of agent were administered to the patient. Each agent holds the potential to determine different cardiovascular toxicities. One of the most common adverse reactions of axitinib is systemic hypertension. A meta-analysis including 77 studies showed that arterial thromboembolism, cardiac ischemia and cardiac dysfunction rate among cardiotoxic effect, and hypertension were the most common and clinically recognized adverse events with OR 5.28 [4.53–6.15] (60). Another meta-analysis showed that the risk of hypertension with axitinib was substantially higher than other approved VEGFR-TKIs. In addition, the risk of all-grade and high-grade hypertension associated with axitinib is significantly higher in RCC than that in non-RCC (61).

Generally, hypertension is an established risk factor for chemotherapy-induced cardiotoxicity, and poorly controlled blood pressure can influence outcomes for cancer patients. Therefore, continuous monitoring and medical treatment with antihypertensive agents are recommended for axitinib-associated hypertension. There is no general consensus on the best modality for blood pressure monitoring. The AIOM (Associazione Italiana di Oncologia Medica-Italian Association of Medical Oncology) guidelines recommend a weekly based monitoring in the first 8 weeks and specifically a blood pressure measurement before every cycle. In addition, guidelines recommend to obtain a good blood pressure before starting treatment (62). The ESMO (European Society of Medical Oncology) recommend generally more frequent BP monitoring in those patients with preexisting hypertension and known to be at higher CV risk. Once stable blood pressure is achieved, the evaluation schedule might be aligned with home BP monitoring or routine clinical evaluations, at least every 2–3 weeks for the remainder of the treatment (63). In this specific case, the patient had history of systemic hypertension and was already on treatment. Home monitoring was then recommended. Despite that increased blood pressure is usually reported after the first dose of treatment, in this case it was observed after the third dose. Therapy combination was used to reach a good blood pressure profile (calcium antagonist was added to angiotensin II receptor blockers).

The second agent administered to the patient was the ICI pembrolizumab. This agent can be associated with a spectrum of adverse effects mainly related to irAEs and can affect multiple organs including the cardiovascular system. Although rare, cardiovascular IrAEs can be fulminant (64). A recent meta-analysis including multiple sources (World Health Organization, WHO pharmacovigilance database with more than 16,000,000 adverse drug reactions, 16 international multi-institutional treatment data, and all published clinical trials to characterize more than 750 fatal irAEs) reported that ICI-associated toxic effects are rare and occur very early after therapy initiation and with marked distinctions between ICI regimens. Combination therapy had more frequent multiorgan involvement, and nearly one-third of all deaths were from myocarditis, myositis, and/or neurologic events (55). Combined PD-1 plus CTLA-4 blockade triggers substantially more irAEs than anti–PD-1 alone (55%–60% vs. 10%–20% high-grade events) (65, 66). Notably neurologic and cardiac toxic effects comprised nearly half of deaths. Many of these cardiological adverse events are often unrecognized until they are severe and potentially fatal. AIOM guidelines suggest to perform an electrocardiogram before treatment. Serial troponin measurements are not recommended as evidence currently does not support their use (67). Troponin evaluation may be considered in those patient candidates to treatment with combination of ICIs known to be more toxic. In case of high-risk treatment, troponin should be repeated at 2, 4, and 12 weeks. Advanced cardiovascular imaging, such as strain analysis by echocardiography (68) and cardiovascular magnetic resonance (CMR) (69), seems a promising tool to detect toxicities and predict outcome, but data are limited and they are not recommended at this stage. Both AIOM and ESMO guidelines recommend, in case of symptoms, to perform comprehensive cardiological evaluations including electrocardiogram, troponin and NT-pro BNP (brain natriuretic peptide) evaluation, echocardiogram with strain analysis, and cardiovascular magnetic resonance (CMR), in case of symptoms and/or troponin level rise and/or ECG changes (5, 62). Of note, guidelines suggest to consider, in addition to chest pain and dyspnea, symptoms such as fatigue and asthenia. Endomyocardial biopsy should be considered if the diagnosis is highly suspected with an otherwise negative workup and/or the patients cannot undergo non-invasive assessment due to hemodynamic instability (17, 70). In this case, the patient was treated with single ICI and serial troponin and/or ECG were then not recommended. She did not present with any toxic effect from pembrolizumab. Troponins and echocardiogram were performed when the patient complained of atypical symptoms, but these did not reveal any abnormalities. In the clinical case presented, we concluded that only axitinib determined the increased value of blood pressure. Therefore, antihypertensive therapy was implemented with a good response preventing further increases.

Anticancer therapies utilized in GU cancer can have cardiac-related toxicities, and the collaboration between oncologist and cardiologist is crucial. One of the priorities of the cardio-oncology field is the possibility to improve the cardiovascular screening to mitigate risk factors for cardiotoxicity prior to the beginning of treatment and to identify high-risk patients requiring a closer follow-up (**Table 1**). The goal is to avoid cancer therapy interruption and to prevent cardiovascular events.

### 3.5 Case 5: Multidisciplinary management of cutaneous toxicities

#### 3.5.1 Case presentation

A 60-year-old male patient with mRCC treated with cabozantinib was referred to our department. His personal history showed type 2 diabetes mellitus, hypertension, and atrial fibrillation.

In July 2012, he underwent surgery of left nephrectomy and histological examination showed renal clear cell carcinoma, pT2a. Therefore, the patient started clinical and radiologic follow-up.

In July 2016, the total body CT scan showed a local relapse of disease and distant metastases, located in the paravertebral muscles, right gluteus muscle, bones, and lungs. In August 2016, the patient underwent a biopsy of the gluteus muscle, which confirmed the diagnosis of metastases from RCC. Therefore, he started first-line therapy with sunitinib 50 mg per day 4 weeks on/2 weeks off, from September 2016 to June 2017. Then, the total body CT scan showed a disease progression to the lungs and muscles. Considering the previous treatment, the patient started therapy with nivolumab and in July 2017 he underwent radiotherapy for muscular metastases (paravertebral and gluteus) and stereotactic radiotherapy on a lung metastasis in July 2020. Nivolumab was administered until February 2021, when the total body CT scan showed a disease progression on the liver and pancreas.

**Figure 6:**
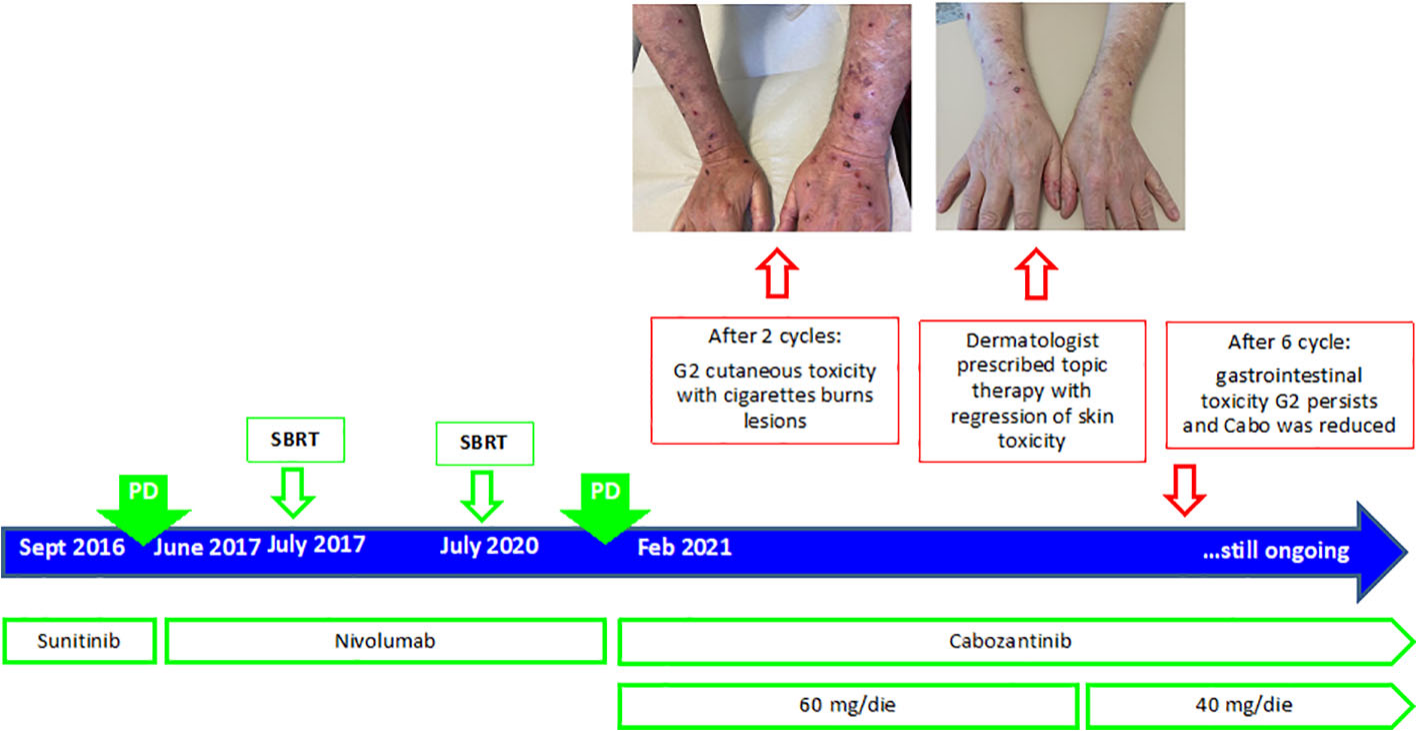
Timeline management of cardiovascular toxicity in the course of ICI- and TKI-based treatments.

At this point, in April 2021, the patient started a third-line treatment with cabozantinib 60 mg daily. After 28 days, at the beginning of the second cycle of therapy, the patient reported erythema on the dorsal hands, not associated with pruritus. However, we decided to continue the therapy. One month later, at the beginning of the third cycle, we found a worsening of cutaneous toxicity, with lesions resembling cigarette burns (**Figure 6**).

At this point, we asked a dermatologic consultant, in order to evaluate and treat these lesions. Since lesions were limited to the upper arms, with less than 30% of skin involved, and Nikolsky sign showed a negative result, we considered this skin eruption as prurigo-like and our dermatologist prescribed azithromycin 1 cp/day for 3 days, fluorescein lotion, silver nitrate gel, cicatrizin gel, zinc oxide, delicate hand cleanser, and nitrile gloves. The patient started this treatment without discontinuation of cabozantinib. At the subsequent visits, the patient showed a reduction in the skin toxicity (**Figure 6**), up to a total regression of the lesions on the hand in July 2021.

However, in August 2021, due to grade 3 gastrointestinal toxicity and an episode of syncope with hypotension, we discontinued treatment with cabozantinib, and a few months later, in October 2021, we decided to resume the cabozantinib at a lower dosage (40 mg).

The total body CT scan, performed in November 2021, showed a partial response of the disease. The patient is still under treatment, with no other severe toxicity.

#### 3.5.2 Dermatologist opinion

Cutaneous adverse events may occur frequently with the use of cabozantinib (71). Cabozantinib is a multi-tyrosine kinase inhibitor (TKI), with activity against MET, RET, AXL, VEGFR2, FLT3, and c-KIT (72). In the METEOR trial, cabozantinib showed a better median PFS and OS versus everolimus in patients who progressed after a previous line with an anti-VEGFR TKI (38, 73). The most frequent adverse events with cabozantinib were diarrhea, fatigue, hypertension, stomatitis, nausea, and hand–foot syndrome.

The hand–foot syndrome (palmar–plantar erythrodysesthesia) is a potentially painful dermatological condition, reported by 43% of the patient in the METEOR trial. The mechanism by which HFS develops is not fully understood; it is possible that the drug interferes with pericyte‐mediated endothelial survival mechanisms, leading to damage to the capillary endothelium in the hands and feet (74) or the inhibition of KIT (strongly expressed in the ductal epithelium of eccrine glands) (75). Prophylactic measures include pedicure to remove hyperkeratosis, use of emollients, topical exfoliation, and protection of pressure‐sensitive areas. For low‐severity cases of HFS, the use of urea cream and clobetasol cream, and analgesics if pain control is needed, may be sufficient to manage the AE (74). Urea cream is recommended as a prophylactic measure with usage from the first day of cabozantinib treatment (76). In our patient, skin involvement was less than 30%; therefore, we decided to continue treatment (77). Overall, the skin toxicity may be due to three different mechanisms of action: immunologic, direct toxicity, or idiosyncrasy. Some skin reactions may also be due to the patient’s comorbidity and drug interaction (78). In the case of ICI-based treatment (anti-CTLA4 and anti-PD1/PD-L1), the reinvigoration of the antitumor T-cell response and the enhanced immunologic activation may result in a variety of autoimmune-like or inflammatory side effects, which can involve almost any organ system, including the skin one (79). Dermatologic complications affect between 30% and 50% of patients on ICIs, and generally, they occur as the earliest events among all irAEs. The most widely reported skin toxicities are maculopapular rash, pruritus, lichenoid eruptions, and vitiligo (80). Although they are most frequently mild and manageable, they significantly impair patients’ quality of life and could lead to treatment interruption. Also, life-threating conditions like Stevens–Johnson syndrome/toxic epidermal necrolysis and the drug reaction with eosinophilia and systemic symptoms (DRESS) may occur (80). In order to avoid severe reactions that can even be lethal for the patient, it is really important to make the right diagnosis very quickly, taking into account appearance and timing and skin involvement, to understand the best pretreatment and or desensibilization, to avoid oncologic treatment discontinuation, and to obtain the best efficacy, a high compliance, and the best quality of life (81, 82). In case of severe reactions (G3–G4 cutaneous toxicity, with diffused eruption), systemic corticosteroids, withholding ICIs, and skin biopsy to exclude other causes and verify the grade of epidermic necrosis should be recommended. ICIs may be reintroduced after the resolution of cutaneous signs (67). However, it depends on clinical evolution. In our case report, the cutaneous toxicity was G2 grade; thus, skin biopsy was not done. A multidisciplinary approach is mandatory in order to create guidelines, considering that each patient is different and can have different reactions; thus, skin toxicity can be cumulative and not predictable in advance. Periodical follow-up, as well as education to an appropriate lifestyle and habits (oncosupportive care: sun protection, emollients, specific shower gel, ideal socks, avoiding aggressive products, etc.) to take care of the skin as a possible indicator of internal disease, is mandatory (83, 84). An appropriate symptomatic and etiologic (when it is possible) treatment is the better strategy for a correct balance. Probably in the future, a genetic analysis will be able to predict personal predisposition and will allow to define personalized treatment, and oncosupportive dermatology will be accepted in each oncologic team. Few biological markers such as rheumatoid factor (RF) greater than 15 IU/ml at baseline and the presence of an HLA-DRB1*11:01 genotype are emerging as potential predictive biomarkers of skin toxicity, especially in case it is associated with pruritus, in patients treated with ICI-based treatment (85, 86). However, further study will be necessary to draw up a detailed algorithm of skin care prevention in mRCC patients to improve the patients’ compliance for both immunological and targeted drugs.

## 4 Discussion and conclusion

Today, most patients with mRCC receive systemic therapy that is ICI- or target-based, alone or in combination with each other, and may develop drug-related symptoms of different grades of severity. With the introduction of novel combinations, there was a dramatic improvement in the outcome of mRCC patients but also the occurrence of adverse events more difficult to manage, as compared with those observed with the previously used TKIs. Furthermore, due to the relatively recent introduction of these combinations in clinical practice, their cumulative dose adverse effects are still unknown. Furthermore, as the immunotherapy may affect any organs, related toxicities are often misunderstood, before becoming from moderate to severe. A prompt recognition and management of these toxicities represents a fundamental issue in oncological clinical practice, since it correlates with the outcome of cancer patients. Although both European and Italian guidelines give well-established protocol to treat immune-related toxicities according to different grades of severity (5, 67), a specific protocol to prevent the risk of developing an adverse event that may lead to a discontinuation of treatment or a dose reduction for mRCC patients has not yet been established.

In this context, MDT evaluations should be provided in any cancer center, especially for those patients, not only the most elderly and fragile, who should be investigated for preexisting unhealthy conditions, which may require a prompt support to finalize their treatment’ program.

In the present paper, we reported a case series of critical toxicities that occurred in our center during treatment for mRCC and a literature review, with the aim of supporting the MDT’s role in genitourinary cancer care. Indeed, the different specialized disciplines integrated in the genitourinary MDT have demonstrated to help oncologists by providing a better care to mRCC patients, mainly during treatment and follow up. Joining the efforts from different healthcare professionals improves patient management, by an early recognition of treatment side effects and relief of severe symptoms that may occur during treatment with both immune- or target-based therapy. In this way, by preventing and reducing drug-related adverse events, patients’ quality of life as well as adherence and compliance to therapies became better (87). According to other complex solid tumors like head and neck cancer (88), we conclude that a comprehensive evaluation and monitoring of mRCC patients by specialized MDTs is strongly recommended to improve treatment adherence and tolerance, reduce long-term side effects, improve quality of life, and ultimately improve treatment outcome and survival.

## Author contributions

MR and MP write the manuscript. All the authors revised and approved the manuscript.
